# Continuous Manufacturing of Solvent-Free Cyclodextrin Inclusion Complexes for Enhanced Drug Solubility via Hot-Melt Extrusion: A Quality by Design Approach

**DOI:** 10.3390/pharmaceutics15092203

**Published:** 2023-08-25

**Authors:** Siva Ram Munnangi, Ahmed Adel Ali Youssef, Nagarjuna Narala, Preethi Lakkala, Sateesh Kumar Vemula, Rohit Alluri, Feng Zhang, Micheal A. Repka

**Affiliations:** 1 Department of Pharmaceutics and Drug Delivery, School of Pharmacy, The University of Mississippi, Oxford, MS 38677, USA; smunnang@go.olemiss.edu (S.R.M.); aayousse@go.olemiss.edu (A.A.A.Y.); nnarala@go.olemiss.edu (N.N.); plakkala@go.olemiss.edu (P.L.); svemula@olemiss.edu (S.K.V.); rkalluri@go.olemiss.edu (R.A.); 2 Pii Center for Pharmaceutical Technology, The University of Mississippi, Oxford, MS 38677, USA; 3 Department of Pharmaceutical Technology, Faculty of Pharmacy, Kafrelsheikh University, Kafrelsheikh 33516, Egypt; 4 College of Pharmacy, The University of Texas at Austin, Austin, TX 78712, USA; feng.zhang@austin.utexas.edu

**Keywords:** cyclodextrin, complexation, hot-melt extrusion, solubility enhancement, quality by design (QbD)

## Abstract

Conventional cyclodextrin complexation enhances the solubility of poorly soluble drugs but is solvent-intensive and environmentally unfavorable. This study evaluated solvent-free hot-melt extrusion (HME) for forming cyclodextrin inclusion complexes to improve the solubility and dissolution of ibuprofen (IBU). Molecular docking confirmed IBU’s hosting in Hydroxypropyl-β-cyclodextrin (HPβ-CD), while phase solubility revealed its complex stoichiometry and stability. In addition, an 11 mm twin-screw co-rotating extruder with PVP VA-64 as an auxiliary substance aided the complex formation and extrusion. Using QbD and the Box–Behnken design, we studied variables (barrel temperature, screw speed, and polymer concentration) and their impact on solubility and dissolution. The high polymer concentration and high screw speeds positively affected the dependent variables. However, higher temperatures had a negative effect. The lowest barrel temperature set near the Tg of the polymer, when combined with high polymer concentrations, resulted in high torques in HME and halted the extrusion process. Therefore, the temperature and polymer concentration should be selected to provide sufficient melt viscosities to aid the complex formation and extrusion process. Studies such as DSC and XRD revealed the amorphous conversion of IBU, while the inclusion complex formation was demonstrated by ATR and NMR studies. The dissolution of ternary inclusion complexes (TIC) produced from HME was found to be ≥85% released within 30 min. This finding implied the high solubility of IBU, according to the US FDA 2018 guidance for highly soluble compounds containing immediate-release solid oral dosage forms. Overall, the studies revealed the effect of various process parameters on the formation of CD inclusion complexes via HME.

## 1. Introduction

Integrating high-throughput screening in drug discovery led to the emergence of lipophilic compounds. However, formulation scientists need help dealing with limited solubility during development and manufacturing. Over 40% of APIs in the market and 70% in the developmental pipeline exhibit poor solubility [1]. Poor solubility may decrease active pharmaceutical ingredients (APIs)’ bioavailability and require higher doses [2]. The bioavailability of poorly water-soluble and high-permeable APIs can be improved by addressing the low aqueous solubility with various pharmaceutical approaches to reduce the dosage and subsequent adverse and side effects. Various approaches, such as size reduction, complexation, salt formation, the use of surfactants, amorphous and crystalline solid dispersions, etc., were adopted by researchers to enhance the solubility and dissolution profile of poorly soluble APIs [3,4]. Among all these strategies, inclusion complexation with cyclodextrin (CD) has been widely investigated and adopted over decades to improve the solubility of poorly water-soluble APIs in the pharmaceutical field. CD is a toroidal-shaped cyclic oligosaccharide molecule with a hydrophilic outer surface and a hydrophobic core. A hydrophobic API becomes hosted in the hydrophobic central cavity of CD. Complex formation depends on the cyclodextrin (CD) cavity and API molecule sizes. The CD cavity must snugly fit the API in its cavity, avoiding undersizing or possessing excessive space. Undersizing of the CD cavity inhibits complexation, while oversizing leads to labile complexes. As a result, the cavity dimensions of CD must be adequately sufficient to allow complex formation, while also limiting the easy escape of the encapsulated molecule from its cavity. Upon adding the API–CD complex to aqueous media, the hydroxy groups on the outer surface of the complex form hydrogen bonds with water molecules, allowing the complex to dissolve [5]. In addition to improving the solubility of the API, the complex formation improves the palatability and stability (e.g., photo and thermal stability) of drugs [6]. The non-covalent interactions during the complex formation between the API and CD enable the dissociation of the API in the presence of aqueous media, thus keeping the complexed and un-complexed molecules of the API in a dynamic equilibrium [7,8]. Various methods were explored for the preparation of CD-based inclusion complexes, for instance, kneading, solvent evaporation, spray drying, electrospinning, microwave irradiation, freeze drying, ball milling, etc.; most of these methods are solvent-intensive processes [9,10,11]. During the last decade, hot-melt extrusion (HME) has been used to prepare many inclusion complexes to enhance the solubility of the API [1,12,13].

HME is a well-established, efficient technology ideally suited for large-scale production, as it facilitates continuous manufacturing with reduced waste and a high yield. HME can produce solid molecular dispersions with numerous advantages over solvent-based techniques such as spray drying and co-precipitation. Thus, it has been extensively adopted and investigated over the last decades to prepare sustained, modified, and targeted drug delivery systems [14]. During the preparation of the solid dispersion, the crystalline API is sheared under controlled heating conditions, and mechanical stress is typically assisted under a thermoplastic polymer, which then becomes dispersed in the polymer matrix either as a crystalline or amorphous form of the API. The intense interactions between the formulation components can alter the properties of the drug, subsequently improving the solubility. 

The drug must be hosted in the central cavity of CD to form the target inclusion complex. However, the conditions for the complex formation are only sometimes achieved during the HME process [12]. Among all the solutions adopted to address this issue, using liquids and polymers to aid the formation of complexes was widely investigated. Manne et al. developed hot liquid-assisted inclusion complexes, wherein the API and CD are mixed with a little solvent and extruded to form a solid API–CD complex [15]. In another study by Thiry et al., the inclusion complex was prepared by adding Soluplus to aid in forming complexes [1]. The drug either dissolves in liquid polymer (miscible) or suspends (immiscible) in a molten matrix to become included in the CD cavity [12]. The use of a polymer not only assists in complex formation through the hot-melt extrusion (HME) process but also acts as a synergistic aid in improving the solubility of the free, unentrapped API and the stability of the API–CD complex. In this case, adding a polymer converts the binary inclusion complex into a ternary inclusion complex (TIC) [16]. The polymer should be hydrophilic, with low viscosity, and possess a glass transition temperature (Tg) significantly below the degradation temperature of the API. 

Quality by design (QbD) promotes a thorough understanding of the potential risks and associated interactions between formulation and process variables through quality risk management and the Design of Experiments (DOE) [17]. The DOE is used to assess the relationship between independent and dependent variables. The Box–Behnken design (BBD) is a statistical optimization design that is commonly used to investigate the impact of formulation and process variables on product quality attributes. BBD is a cost-effective and time-saving statistical design because it involves fewer experimental trials than other statistical designs [18,19].

The current study was intended to statistically evaluate the effect of various process and material variables on the solubility enhancement and in vitro dissolution performance of ternary inclusion complexes (TIC) produced by the HME process. Ibuprofen (IBU) was chosen as a model API because of its low solubility (0.076 mg/mL at 25 °C) and high permeability (log P—3.7) [20]. PVP VA-64 was chosen as the polymer forming matrix in this study because this polymer exhibited good affinity toward IBU in many earlier published investigations [21,22]. The stoichiometry of the complexation between IBU and CDs was investigated using molecular docking and phase solubility studies. Furthermore, the effects of polymer concentration, extrusion speed, and temperature on the solubility and dissolution of IBU were studied using the BBD–QbD model. Moreover, the prepared complexes were evaluated for saturation solubility, in vitro dissolution, crystallinity, thermal degradation, and compatibility among the formulation components. The overall objective of this study is to provide a better understanding of the critical attributes involved in the continuous manufacturing of CD inclusion complexes through HME. The successful establishment of this technology transforms the conventional techniques, which are associated with several disadvantages, into a more scalable and efficient manufacturing technique.

## 2. Materials and Methods

### 2.1. Materials

Ibuprofen (IBU) and Kollidon VA-64 (PVP VA-64) were kind gift from BASF. Ashland kindly gifted the α-CD, β-CD, γ-CD, HPβ-CD, and HPγ-CD. Transparent hard gelatin capsule shells, size 00, were purchased from Total Pharmacy Supply Company (Arlington, TX, USA). Methanol and other chemicals were purchased from ThermoFisher Scientific (Waltham, MA, USA).

### 2.2. High-Performance Liquid Chromatography (HPLC) Method

Ibuprofen was analyzed using the HPLC method under the following conditions: Column-Waters C18 250 × 4.60 mm; flow rate—0.625 mL/min; wavelength—263 nm; and mobile phase—a mixture of methanol and 0.4% glacial acetic acid in the ratio of 85:15. The 20 µL samples were injected into Waters e2695 HPLC equipped with a Waters 2489 UV/Visible detector.

### 2.3. Determining the Stoichiometry of the Binary Complex

#### 2.3.1. Molecular Docking Studies

Autodock Vina molecular modeling simulation software v1.5.6 (Scripps Research, general public license) investigated the molecular interaction between IBU and selected CDs. PyMol 2.3.4 (Schrödinger, open-source foundation) was used to convert the three-dimensional structure of IBU, α-CD, β-CD, γ-CD, HPβ-CD, and HPγ-CD into PDB files. The conformation setting for the ligand IBU was flexible with rotatable bonds. At the same time, the receptor or host α-CD, β-CD, γ-CD, HPβ-CD, and HPγ-CD were rigid, with the rest of the parameters in Autodock Vina being set to default. The docking pose with the highest binding affinity (lowest binding energy) was considered the lead CD for the complex formation [23,24].

#### 2.3.2. Phase Solubility Studies

Excess IBU was added to 10 mL of CD solutions with concentrations ranging from 0.5 to 15 mM. Then, the drug–CD mixtures were agitated on an orbital shaker for 48 h at 25 ± 2 °C under continuous shaking until equilibrium was achieved. Samples (1 mL) were collected, centrifuged using AccuSpin 17R centrifuge (Thermo Fisher Scientific, Waltham, MA, USA) at 13,000 rpm for 15 min, and filtered through a 0.22 µm polyvinylidene fluoride (PVDF; MilliporeSigma St. Louis, MO, USA) membrane filter before being analyzed using the HPLC method described above. The experiment was performed in triplicate. The apparent stability constants (*Ks*) of the binary inclusion complex were calculated using the slope of the molar concentration of dissolved IBU versus the molar concentration of CD plots based on the following Equation (1) [25].

(1) $$K_{s}=\frac{slope}{s_{0\;}(1-slope)}$$

where *S*_0_ is the solubility of IBU in the absence of cyclodextrin (intercept). The complexation efficiency (*CE*) and the ratio of complexation between IBU and CDs can be calculated using the following Equations (2) and (3) [25]:

(2) $$CE=\frac{slope}{1-slope}=s_{0}\times K_{s}$$

(3) DCD=11+1CE

where *D* is the concentration of free IBU, and *CD* is the concentration of free cyclodextrin.

### 2.4. Preparation of Samples

#### 2.4.1. Ternary Inclusion Complex (IBU/HPβ-CD/PVP VA-64) Preparation Using QbD

##### Preliminary Studies

Developing a successful product using HME technology is primarily based on selecting an appropriate process and formulation parameters, including screw speed, processing temperature, polymer, and extrusion concentration. The polymer was selected based on many earlier published studies [21,22]. The QbD model’s upper and lower polymer concentration limits were screened between 5% and 50% *w*/*w*. At the same time, the extrusion temperature and screw speed were screened between 115 and 145 °C and 10 and 40 rpm, respectively. All other processing parameters were adjusted based on our earlier reported studies. For the studies, IBU and HPβ-CD were maintained at stoichiometric ratio of 1:1. Before extrusion, the physical mixtures were blended in a V-blender for 10 min at 10 rpm. An 11 mm twin-screw extruder (Process 11, Thermo Fisher Scientific, Austin, TX, USA) was used for extrusion with a standard configuration comprising 3 mixing zones to ensure proper components’ distribution. The feeding rates were 2, 3, and 4 rpm for 15, 30, and 45 rpm screw speeds, respectively.

##### Box–Behnken Design (BBD)

Response Surface Methodology (RSM) is a collection of sound statistical and mathematical tools for developing, improving, and optimizing processes and/or products. The field of RSM consists of an experimental strategy for discovering the design space of the process or independent factors and empirical statistical modeling to find a suitable approximating relationship between the response and the process variables, thus providing optimization methods for exploring the values of optimum levels for the process variables that yield desirable values for the target response. BBD is a class of rotatable or nearly rotatable second-order designs comprising at least three-level incomplete factorial designs [26]. The main advantage of the BBD is that the statistical design does not contain combinations in which all independent variables are simultaneously at their highest or lowest corresponding levels. Therefore, the design helps escape experiments performed under extremely unnecessary conditions, where unacceptable results might occur [26]. Thus, BBD could determine the potential interactions between selected parameters and avoid the time-consuming optimization process by reducing the number of experimental runs. Accordingly, the extruded complexes were optimized by the RSM “Box–Behnken design; BBD” using Design-Expert software (StatEase Inc., Minneapolis, MN, USA, Version 13.0). Three independent variables were employed to find the optimal levels for the mean solubility (mg/mL, Y_1_) and release after 30 min (%, Y_2_) at 3 levels, as provided by the statistical design. The selected variables were the PVP VA-64 concentration (X_1_, % *w*/*v*), extrusion temperature (X_2_, °C), and screw speed (X_3_, rpm), which were explored at three different levels: one central point (X_1_; 20% *w*/*v*, X_2_; 130 °C, and X_3_; 30 rpm), level +1 (X_1_; 30% *w*/*v*, X_2_; 145 °C, and X_3_; 45 rpm), and level −1 (X_1_; 10% *w*/*v*, X_2_; 115 °C, and X_3_; 15 rpm). The details of the applied BBD design are provided in Table 1 and Table 2. A total of 17 experimental runs were tested, including 12 bifactorial points (levels; 0 and ±1) and 5 central point replicates for estimation of pure error estimation. However, only 14 runs were successful, and the other 3 experimental runs failed to extrude. All experimental runs were haphazardly conducted to ensure minimal effects of variability within the observed responses due to systematic errors. The extruded material was milled using a coffee blender and passed through US mesh #30.

#### 2.4.2. Binary Inclusion Complex

The HPβ-CD was dissolved in a mixture of isopropanol and water (40:60). IBU was then added to the HPβ-CD solution (IBU and HPβ-CD in 1:1 molar ratio) under continuous magnetic stirring at 600 rpm for 6 h until a clear solution was attained. Next, the organic solvent was removed using BUCHI rotavapor (Rotavapor R-100, BUCHI Corporation, DE, USA). Then, the aqueous solutions were subjected to overnight freezing (−80 °C) in a So-Low Ultra-Low Freezer (Environmental Equipment & Services, Inc., Cincinnati, OH, USA). The samples were then lyophilized using a Labconco FreeZone shelf freeze drier (Labconco, Kansas City, MO, USA) for 36 h [27]. The shelf temperature was increased and maintained at −20 °C for 8 h at a pressure of 0.05 mbar during the primary drying stage. Then, shelf temperature was again increased and maintained at 0 °C for 12 h at a heating rate of +1 °C/min. During the secondary drying, the shelf temperature was increased to 25 °C at +1 °C/min and maintained for 16 h at 25 °C.

#### 2.4.3. IBU-PVP VA-64 Solid Dispersion

The solid dispersion of IBU-PVP VA-64 was prepared with a drug content of 9.4% *w*/*w*, identical to optimized TIC, wherein, for solid dispersion, the CD in TIC was replaced by a polymer. The IBU and PVP VA-64 mixture was blended for 10 min at 10 rpm in a V-blender. An 11 mm twin screw extruder was used with a screw configuration resembling that used in TIC preparation. The extrusion was carried out at the same parameters for optimizing TIC formation.

### 2.5. Saturation Solubility

In glass vials containing 20 mL of distilled water, excesses of pure IBU, IBU–HPβ-CD, IBU-PVP VA-64, TIC-PM, and TIC were added. The vials were left on an orbital bio-shaker for 48 h at 25 °C ± 2 °C with continuous stirring at 400 rpm to achieve equilibrium. After 48 h, the appropriate aliquots of the sample were withdrawn, 0.22 µm PVDF filtered, and analyzed using HPLC.

### 2.6. Attenuated Total Reflection (ATR)

The IR spectra of IBU, HPβ-CD, PVP VA-64, physical mixture, solid dispersion, and binary and TIC of IBU were determined using Agilent Cary 660 FTIR spectrometer (Agilent Technologies, Santa Clara, CA, USA). A small sample was added to the diamond crystal and compressed with a Miracle high-pressure clamp. The sample was analyzed over a 4000–200 cm^−1^ scanning range with a 4 cm^−1^ resolution. FTIR spectrometer was equipped with attenuated total reflection (Pike Technologies, Madison, WI, USA), a single bounce, and a diamond-coated ZnSe internal reflection element.

### 2.7. Differential Scanning Calorimetry (DSC)

DSC measurements of IBU, HPβ-CD, PVP VA-64, physical mixture, solid dispersion, and binary and TIC of IBU were performed using a Discovery DSC 25 instrument (TA Instruments, Newcastle, DE, USA) equipped with a refrigerator cooling system (RCS90). A sample of ~5 mg was measured and taken into an aluminum pan. The samples were scanned at a steady rate of 10 °C per minute over a 0–150 °C temperature range using a nitrogen purge of 50 mL/min against the empty pan as reference.

### 2.8. X-ray Diffraction (XRD)

The crystallinity of IBU, HPβ-CD, PVP VA-64, physical mixture, solid dispersion, and binary and TIC of IBU was evaluated by PXRD analysis. The diffractograms were captured with a Rigaku X-ray system (D/MAX-2500PC, Rigaku Corp., Tokyo, Japan) using Cu rays (λ = 1.54056 Å) at 40 kV and 40 mA over a 2–50° scanning range with a step width of 0.02°/s and a scan speed of 0.02 s.

### 2.9. ^1^H Nuclear Magnetic Resonance (NMR)

IBU, HPβ-CD, PVP VA-64, physical mixture, solid dispersion, and binary and TIC of IBU were recorded on a Bruker Advance DRX 500 MHz FT NMR instrument at 298 K. The exact weight of each sample was added and dissolved in DMSO-d_6_, which is used for recording the ^1^H NMR. The spectra were processed with MestRenova 14.3.1 software.

### 2.10. Drug Content Uniformity

The milled extrudates containing 20 mg of IBU (for TIC with 30% polymer—242 mg of the blend, 20% polymer—212 mg of the blend, and 10% polymer—188 mg of the blend) were added to 100 mL mobile phase and sonicated for 30 min in Branson 2510 bath sonicator (Branson Ultrasonic Corp., Danbury, CT, USA). The 2 mL sample was filtered using a 0.45 μm Nylon membrane filter into a vial and diluted 10 times using mobile phase. Later the diluted sample was analyzed via HPLC.

### 2.11. In Vitro Dissolution Studies

Using a USP dissolution apparatus type I (Basket), in vitro dissolution tests were performed on pure IBU, IBU–HPβ-CD, IBU-PVP VA-64, TIC-PM, and TIC. The dissolution medium was 500 mL of 0.1 N HCl and pH 7.2 phosphate buffer. The pH 7.2 phosphate buffer was used to dissolve IBU according to USFDA specifications, whereas 0.1 N HCl was used to differentiate drug release between different formulations. Pure IBU and inclusion complexes equivalent to 20 mg of IBU were filled into gelatin capsules (size 0). They were added to the dissolution medium, which was kept at a constant temperature of 37 ± 0.5 °C and a speed of 100 rpm throughout the study. The 500 mL quantity was chosen as it can dissolve the 20 mg of IBU, considering the saturation solubility of 0.076 mg/mL at pH 1.2 (0.1 N HCl) [28]. A 5 mL sample was withdrawn at predetermined intervals of 5, 10, 15, 20, 30, 45, and 60 min, and the same quantity of fresh media was replaced to maintain sink conditions. The collected samples were filtered with a 0.45 syringe filter, and HPLC was used to analyze them to calculate the percentage of drug release.

The percentage of drug released over time was graphed and used to identify the percent dissolved after 30 min (PD30). The initial dissolution rate (IDR) was also determined by calculating the percentage of drug dissolved per minute during the first 30 min [29]. Dissolution efficiency (DE) was measured using the trapezoidal rule to find the area under the dissolution curve at a given time, expressed as a percentage of the area of the rectangle described by 100% dissolution at the same time [30]. Lastly, the relative dissolution rate (RDR) was calculated as the ratio between the amount of drug dissolved from the best formulation (TIC) and that dissolved from the pure IBU after 30 min [31]. 

### 2.12. Stability Studies

The milled-extrudates-filled capsules were packed into a High-Density Polyethylene (HDPE) bottle and were subjected to accelerated stability conditions (40 ± 2 °C/75 ± 5% RH) for 3 months (90 days) according to ICH guidelines [32]. The samples were studied for changes in drug content, dissolution, and recrystallization using DSC.

## 3. Results and Discussion

### 3.1. Determining the Stoichiometry of the Binary Complex

#### 3.1.1. Molecular Docking Studies

**Figure 1:**
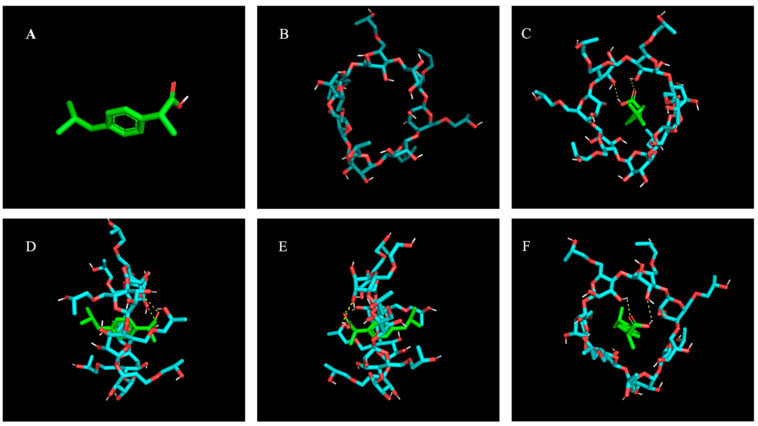
Molecular docking studies of IBU and HPβ-CD:IBU (**A**), HPβ-CD (**B**), complex front view (**C**), complex side view (**D**,**E**), and complex back view (**F**).

The complexation mechanism underlying the hosting of IBU in α-CD, β-CD, γ-CD, HPβ-CD, and HPγ-CD was investigated by molecular docking studies [24]. Figure 1 shows the optimal structures of the IBU/HPβ-CD inclusion complex. The free binding energy of IBU with α-CD, β-CD, γ-CD, HPβ-CD, and HPγ-CD was found to be −3.7 Kcal/mol, −4.4 Kcal/mol, −4.2 Kcal/mol, −5.4 Kcal/mol, and −3.8 Kcal/mol, respectively. Among all investigated cyclodextrins, the results showed that IBU was hosted in the hydrophobic cavity of HPβ-CD with high binding affinity. The hollow hydrophobic cavity of HPβ-CD was discovered to be sufficient to accommodate the entire molecule of IBU. Therefore, the inclusion complex with HPβ-CD was selected to prepare TIC and was further evaluated. Furthermore, the formation of two hydrogen bonds (yellow dotted lines in Figure 1F) in the IBU/HPβ-CD inclusion complex was revealed by detailed analysis.

#### 3.1.2. Phase Solubility Studies

**Figure 2:**
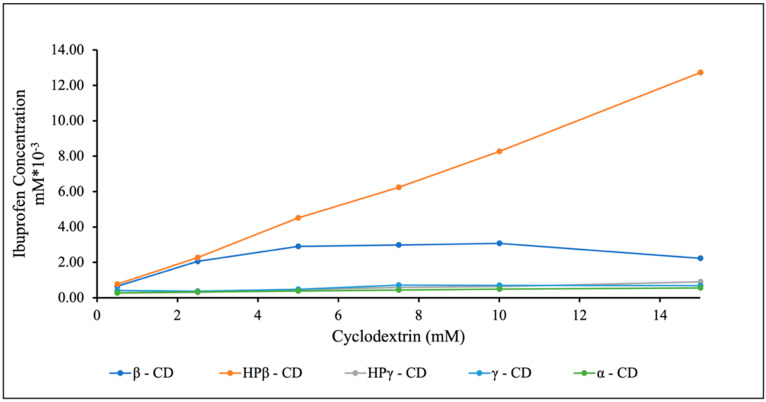
Phase solubility study graphs between IBU and different CDs.

The driving force for inclusion complexation entails the following: 1. eliminating hydrophobic cavity-bound high-energy water and developing van der Waals forces; 2. hydrophobic interactions; 3. electrostatic interactions; 4. hydrogen bond interactions; 5. charge-transfer interaction [33]. The phase solubility diagrams of IBU-CD are graphically illustrated in Figure 2, which shows a linear increase in IBU solubility with HPβ-CD concentration. According to Higuchi and Connors’ theory, the obtained curves are A_L_-type phase solubility [34]. This curve represents the formation of inclusion complexes in the stoichiometric molar ratio of 1:1 between IBU and HPβ-CD [4]. The value of Ks for binary inclusion complex was found to be 3279.6 M^−1^. Typically, the value of Ks varies with the ratio of reacting species; for example, Ks ranges from 10^2^ to 10^3^ M^−1^ and is essentially not more than 10^4^ M^−1^ [35]. The higher the Ks is, the more stable and better the host–guest complexation efficiency is. The CE of the binary inclusion complex was observed to be 0.820 [36]. The findings are consistent with those obtained from in silico molecular docking studies, where the free binding energy of IBU with HPβ-CD was the highest compared to other cyclodextrins. In the case of other cyclodextrins, IBU with β-CD and γ-CD followed a B_S_-type phase solubility curve. In contrast, IBU with α-CD and HPγ-CD followed an A_L_-type phase solubility curve, but the magnitude of solubility improvement was minute compared to that of HPβ-CD. The Ks and CE values of all the CDs are represented in Table S1.

### 3.2. QbD

#### 3.2.1. Preliminary Studies

Yani et al. studied IBU’s miscibility with PVP VA-64, Eudragit EPO, and hydroxypropyl methyl cellulose (HPMC). The molecular simulation studies demonstrated that IBU has high miscibility with PVP VA-64 and Eudragit EPO compared to HPMC, reflected in the dissolution studies with drug release of more than 85% within 15 min. The H-bond interaction was found to be higher in IBU/Eudragit EPO followed by IBU/PVP VA-64; however, the H-bond lifetime between IBU/PVP VA-64 was 1.5-fold higher compared to IBU/Eudragit EPO. These results demonstrated the formation of a more stable H-bond interaction with PVP VA-64 compared to Eudragit EPO [21]. In another study, Tabriz et al. developed 3D-printed tablets of IBU with PVP VA-64, Eudragit EPO, and Soluplus. The dissolution studies revealed that PVP VA-64 inhibited the recrystallization of IBU compared to the other two polymers [22]. Moreover, PVP VA-64 is a polyvinylpyrrolidone-based polymer that is well-established as a solubilizer and crystallization inhibitor while preparing many solid dispersions [37]. Thus, with the primary objective of improving solubility and inhibiting recrystallization of any free uncomplex IBU, PVP VA-64 was selected as the best candidate for this study with its dual mechanism for serving the objective.

The polymer concentration was screened between 10% and 30% *w*/*w*. A polymer concentration below 5% resulted in high torque, halting the extrusion process, while a concentration above 30% *w*/*w* did not significantly improve IBU solubility or the dissolution rate compared to a 30% *w*/*w* polymer concentration. The extrusion temperature ranged from 115 to 145 °C, since 115 °C is required to liquefy the polymer (for PVP VA-64 the loss tangent (tan δ) = 1 at 115 °C), while 145 °C was chosen as the upper limit because IBU tends to evaporate above 150 °C [38,39,40,41]. The screw speed was selected between 15 and 45 rpm since the higher screw speed (>50 rpm) resulted in a high torque >70%.

#### 3.2.2. Formulation Development Using BBD

BBD was applied to study the main and interaction effects of the selected independent variables on the dependent variables based on the preliminary trials described above. BBD was also used to obtain second-order polynomial equations and construct contour plots to predict the target responses. BBD provided fewer experimental runs (17) than a complete factorial design (27) in this study. The number of experimental runs (N) required for BBD is defined as N = 2k (k − 1) + C_0_, (k is the number of independent variables, and C_0_ is the number of central point replicates). The randomized experimental runs with levels (software and actual) and the corresponding response values obtained for solubility and release are presented in Table 2.

**Figure 3:**
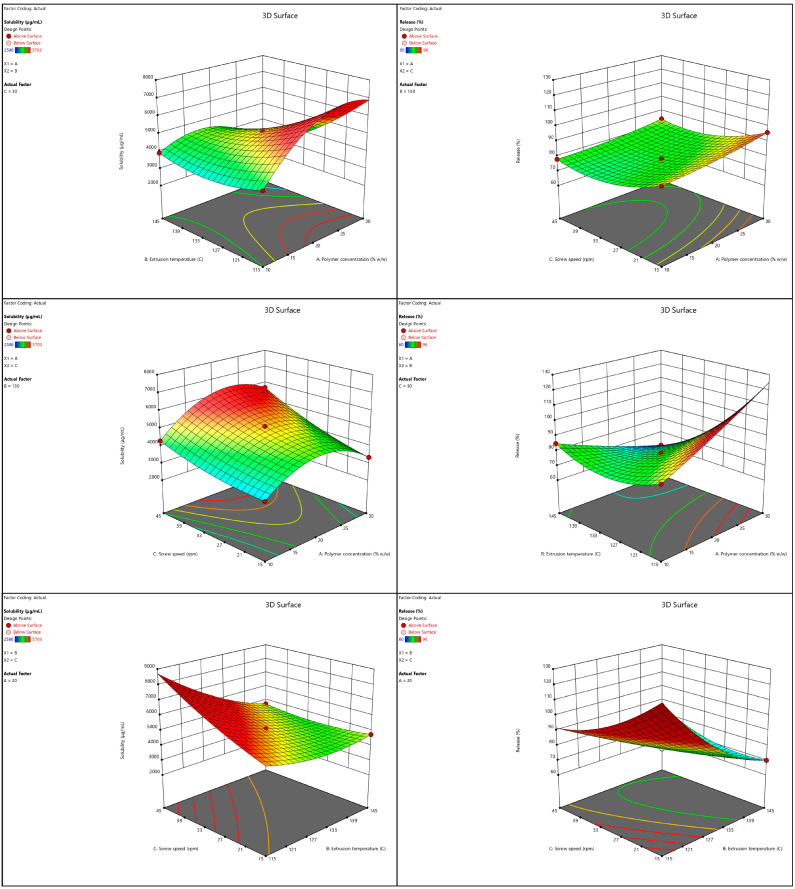
Response surface 3D plots showing the effect of the independent variables on solubility and release of hot-melt extruded TIC.

The fit statistics were calculated from the sequential model comparison to choose the best statistical model for fitting between different suggested software models. Analysis of variance (ANOVA) was conducted for each fitting model to analyze the significance of each model term along with the interaction among different model terms (Table 3). The model F value, standard deviation, mean, and coefficient of variation were calculated for each response. Three-dimensional and interaction plots were generated to study and evaluate the main and interaction effects on the selected responses, as illustrated in Figure 3.

#### 3.2.3. Effect of Independent Variables on IBU’s Solubility and Dissolution

The statistical results of the selected quadratic models were strong—R^2^ = 1.0 for Y_1_ and R^2^ = 0.9999 for Y_2_—revealing a significant relationship between the screened independent variables and their corresponding dependent variables at a 95% confidence level. ANOVA analysis revealed that linear mixture parameters significantly affected drug solubility (*p* < 0.0001). The results also showed that drug solubility was significantly affected by the synergistic effect among the tested independent variables (Table 3). The model signal-to-noise ratio (adequate precision > 4) demonstrated a satisfactory signal to navigate the software design space. The following quadratic model equations can explain the effect of coded factors on drug solubility and dissolution as a function of the independent factors.

IBU solubility mgmL     =5131+377.5∗X1−1120∗X2+855∗X3−1038∗X1X2+325.5∗X1X3−871∗X2X3     −1239.25∗X12+474.75∗X22+253.25∗X32

$$\begin{array}{c} IBU\;Release\;\left(\%\right)\;at\;30\;min\\ \;=78.02+3.25*X_{1}-15.75*X_{2}+4.75*X_{3}-15.75*X_{1}X_{2}-0.25*X_{1}X_{3}+17.25*X_{2}X_{3}\\ \;-1.135*X_{1}^{2}+11.365*X_{2}^{2}+8.865*X_{3}^{2} \end{array}$$

The polynomial equation can predict the solubility (mg/mL) and release (%) values with any given level of each independent variable. The sign and magnitude of the variable coefficients in the regression equations are used to understand the effect of their corresponding terms. Any equation term with a positive coefficient explains that an increase in the term results in a simultaneous increase in the respective response, whereas a negative coefficient shows the opposite [42].

#### 3.2.4. Effect of Independent Factors on Saturation Solubility

According to the solubility equation, a positive trend in solubility was observed with a high polymer concentration, low processing temperature, and high screw speed. The formation of a TIC among IBU, HPβ-CD, and PVP VA-64 may be responsible for the increase in the apparent solubility of the drug. PVP VA-64 has an amphiphilic structure with a hydrophilic polyvinylpyrrolidone group and a lipophilic vinyl acetate group. The improvement in the aqueous solubility might be attained in two ways: (1) the hydrophilic part of the polymer forms molecular assemblages with the IBU–HPβ-CD complex creating water-soluble complexes, and (2) the lipophilic part of the polymer interacts with the free IBU, while the hydrophilic part improves the wettability [37]. The temperature has a minimal effect on solubility enhancement. This outcome could be because all extrusion temperatures are above the melting points of IBU and the Tg of the polymer. At all these temperatures, the drug melts and becomes miscible with the polymer, creating an ideal environment for IBU encapsulation into HPβ-CD [12]. Except for the F14 formulation with 10% polymer, all the formulations (F4, F7, and F12) with more than 10% polymer and an extrusion temperature of 115 °C resulted in high torque during extrusion, halting the process. The temperature of 115 °C (very close to the Tg of the polymer) was associated with high polymer concentrations and resulted in the formation of a highly viscous, softened polymer that formed a solid mass of material buildup around the mixing zones in the barrel, increasing the torque [43]. The formulations with a low polymer concentration, i.e., a 10% polymer concentration, resulted in fine and powdery extrudate compared to the brittle and large granular extrudates of formulations with a higher polymer concentration. Regarding the screw speed, the 3D surface plots (Figure 3) revealed that when PVP VA-64 was used at the lowest concentration and minimal screw speed, it resulted in lower solubility values, per previously reported studies [18].

#### 3.2.5. Effect of Independent Factors on Dissolution

The polymer concentration, temperature, and screw speed had a similar effect on dissolution as they did on solubility. Increasing the polymer concentration, decreasing the temperature, and increasing the screw speed showed a positive trend in dissolution. An increased polymer concentration improved solubility, which resulted in a faster dissolution rate. The temperature had the same minimal effect on dissolution as on solubility. Low temperature and low screw speeds were associated with high polymer concentration and favored faster dissolution rates. This outcome could be because temperatures above those of the polymer’s Tg and the drug’s Tm, combined with high screw speeds, i.e., a reduced residence time and a high polymer concentration, are sufficient for even polymer distribution and the production of crumbling and hard granules, resulting in faster dissolution rates [44].

#### 3.2.6. Optimization and Validation Trials

**Figure 4:**
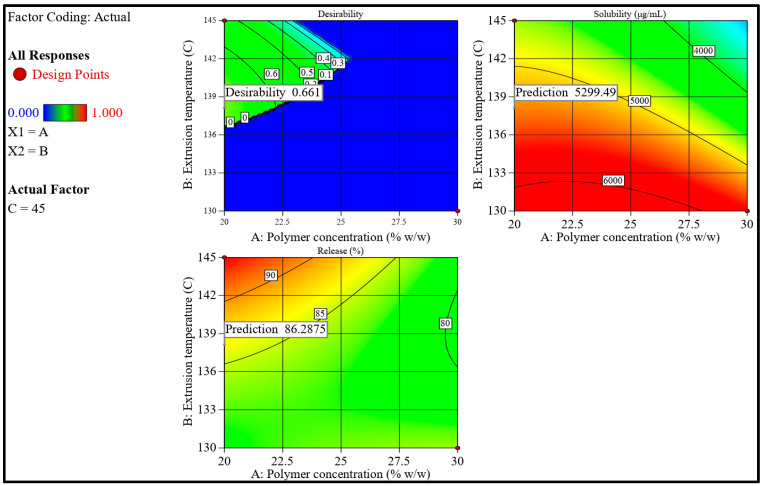
Contour plots showing the desirability and predicted response for the software-proposed solution based on the criteria of the optimization step.

After building up good regression models, the next step toward optimal statistical analysis is the numerical optimization stage, to achieve the study goals by selecting the level of factors. The extruded complexes were optimized by setting the independent variables’ goals and study responses and applying the global desirability function (D), as provided in Figure 4 and Table 4.

The lower and upper limits of PVP VA-64 were set at 20% and 30% *w*/*w*, respectively, to minimize concentration but avoid the fine and powdery granules that were reported with 10% *w*/*w* PVP VA-64. The temperatures for extrusion were chosen to be between 130 and 145 °C. Since CD is a non-thermoplastic component of the formulation, the molten polymer and drug must provide adequate lubrication during extrusion. As the lower temperature of 115 °C is very close to the Tg of the polymer, with higher concentrations of polymer (i.e., >10%) resulting in higher viscosity, which is unable to provide sufficient lubrication for the extrusion, resulting in the halting of the extrusion process with high torque. The temperature goal was set to maximum to have the least resistance during the extrusion process (i.e., less torque). Finally, the screw speed limits were set in the same manner as in BBD. According to the software, 22 solutions were proposed to achieve the required responses with 95% confidence intervals (CI) (Table 5). One formulation with a polymer concentration of 20% *w*/*v* was extruded at 138.1 °C with a screw speed of 45 rpm to fulfill the optimum formulation requirements (Table 5). Using these selected variables and their respective levels was predicted to prepare a complex formulation with an improved solubility and a release percentage of 5299.706 mg/mL and 86.286%, respectively. The suggested solution is graphically illustrated by the interaction plots shown in Figure 4. A validation trial (TIC) was extruded in triplicate to compare the observed values against their respective software-predicted values. The mean practical solubility and release values were within 95% CI of the software-predicted values, as shown in Table 6.

### 3.3. Saturation Solubility

The solubility of pure IBU in water was approximately 0.076 mg/mL. IBU has a pH-dependent solubility, with a basic pH having the highest solubility. Binary inclusion complexes (IBU–HPβ-CD) were 56 times more soluble in water than in pure IBU. The solubility of IBU in the solid dispersion (IBU-PVP VA-64) was 38-fold greater than that of pure IBU. At the same time, TIC-PM increased IBU solubility almost identically to the solid dispersion, with a 37-fold increase compared to pure IBU. The optimized TIC improved IBU solubility 69-fold compared to pure IBU, which is higher than the binary inclusion complex, the solid dispersion, and TIC-PM. In the case of the binary complex, the hostage of IBU in the interior cavity of HPβ-CD may have contributed to the increased solubility. While in the solid dispersion, the amorphous conversion of IBU by molecularly dispersing it in the polymer matrix would be the reason for solubility enhancement. In TIC, the formation of H-bonds between hydroxyl groups of the polymer and HPβ-CD might have formed molecular assemblages, influencing the solubility enhancement of IBU. As a result, amorphization, CD’s hydrophilicity, and improved complexation and stability efficiency by incorporated polymers were attributed to the increased solubility of IBU by TIC [24].

### 3.4. ATR

**Figure 5:**
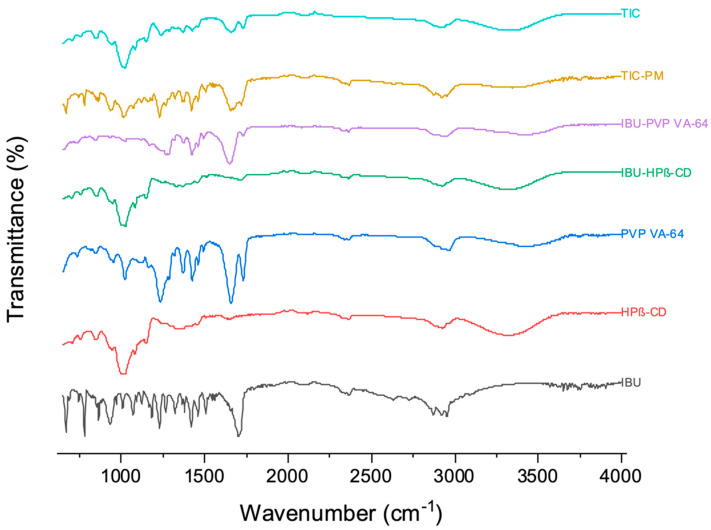
IR spectra of pure IBU, HPβ-CD, PVP VA-64, IBU–HPβ-CD (binary complex), IBU-PVP VA-64 (solid dispersion), TIC-PM (physical mixture), and TIC (ternary inclusion complex).

Infrared spectroscopic bands of IBU, as shown in Figure 5, revealed a typical O-H stretching of carboxylic acid and C-H alkane stretching between 2850 and 3000 cm^−1^, C=O stretching at 1720 cm^−1^, and C-H bending at 770 cm^−1^ [45]. The HPβ-CD peaks showed broad bands between 3300 and 3400 cm^−1^, corresponding to the stretching vibrations of -OH groups caused by intermolecular H-bonds, while the bands at 2937.51 cm^−1^, 1161.37 cm^−1^, and 1043.24 cm^−1^ corresponded to C-H stretching, C-H vibrations, and C-O stretching [45]. PVP VA-64 contained two hydrogen bond acceptor groups derived from the pyrrolidone ring’s carbonyl group (at 1690 cm^−1^) and vinyl acetate (at 1780 cm^−1^). It also showed peaks between 2900 and 3500 cm^−1^, owing to more O-H groups. It also revealed a band at 1270 cm^−1^ attributed to aromatic amine C-N stretching [46]. The IR bands of the binary complex (IBU–HPβ-CD) prepared by freeze drying revealed a missing C=O stretching of IBU at 1720 cm^−1^, confirming IBU’s inclusion in the hydrophobic cavity of HPβ-CD, forming a binary complex. The solid dispersion formed by IBU-PVP VA-64 showed all the IBU peaks but with less intensity than the pure IBU spectrum, attributed to the low concentration of IBU in the dispersion. It also showcased a broad peak ranging from 1650 cm^−1^ to 1800 cm^−1^, which could be the sum of the carbonyl peaks in IBU and PVP VA-64. The IR spectra of TIC-PM was discovered to be a sum of all the materials’ IR spectra. TIC formed by HME had a lower peak intensity between 1650 cm^−1^ and 1800 cm^−1^, which could be attributed to the low amount of PVP VA-64 and IBU dispersed in HPβ-CD, or the peaks could only belong to PVP VA-64, with the carbonyl peak of IBU disappearing by forming a complex with HPβ-CD, as seen in the binary complex. The loss of the crystallinity of IBU, as seen from the data obtained in calorimetry and X-ray crystallography, strongly supports the latter (complex formation). It is worth noting the disappearance of C-H alkane stretching (2850–3000 cm^−1^) in IBU–HPβ-CD, IBU-PVP VA-64, and TIC, which could be attributed to the H-bond formation between IBU and other excipients [46]. No new bands were observed in any of the IR spectroscopic graphs, thus confirming that there was no covalent interaction between IBU and the excipients. The three-month accelerated stability studies represented in the supplementary files revealed the stability of optimized TIC without any interaction with other excipients.

### 3.5. DSC

**Figure 6:**
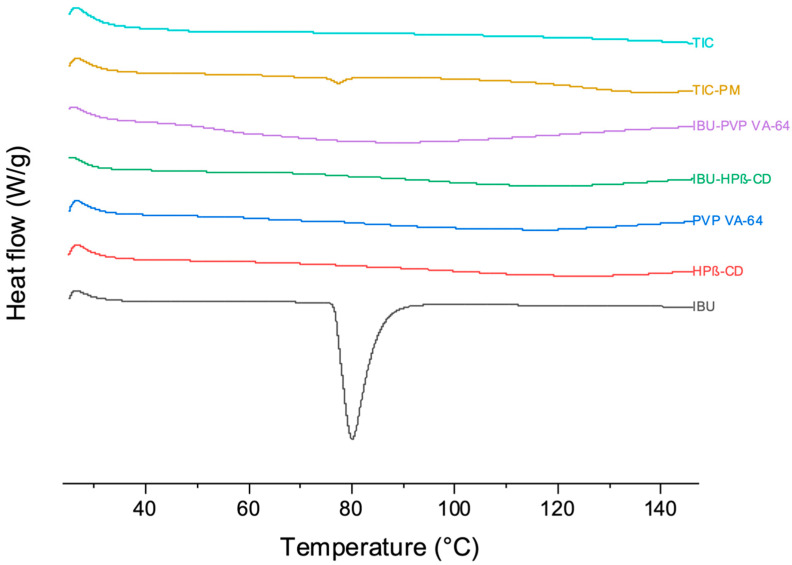
DSC thermograms of pure IBU, HPβ-CD, PVP VA-64, IBU–HPβ-CD (binary complex), IBU-PVP VA-64 (solid dispersion), TIC-PM (physical mixture), and TIC (ternary inclusion complex).

The thermal properties of IBU, the excipients, and the formulations were analyzed using DSC, which provides an inclusive physicochemical status of the guest molecule within the cyclodextrin cavity. The absence or shifting of the endothermic peak of molecules may indicate changes in the crystal lattice, melting point, boiling point, or sublimation. As represented in Figure 6, the calorimetric graphs of IBU revealed a prominent and sharp endothermic peak at ~80 °C, corresponding to the melting point (T_m_) of pure IBU [47]. Due to its amorphous nature, HPβ-CD and PVP VA-64’s calorimetric graphs have no prominent endothermic peak. The solid dispersion of IBU-PVP VA-64 displayed the absence of a sharp endothermic peak of IBU. Instead, a strong and broad endothermic peak (T_g_) was observed between 60 and 100 °C, indicating the amorphous conversion of IBU.

Similarly, no endothermic peak of IBU was observed in the binary complex IBU–HPβ-CD. TIC-PM exhibited an endothermic IBU (T_m_) peak at ~80 °C but with low intensity due to the low concentration and dispersal of IBU in the physical mixture. The absence of an endothermic peak for IBU in TIC confirmed its amorphous conversion during the HME process, predominantly by including IBU in the central cavity of HPβ-CD [20]. The TIC formulation was found to be stable without any recrystallization of IBU after three months under accelerated stability conditions.

### 3.6. XRD

**Figure 7:**
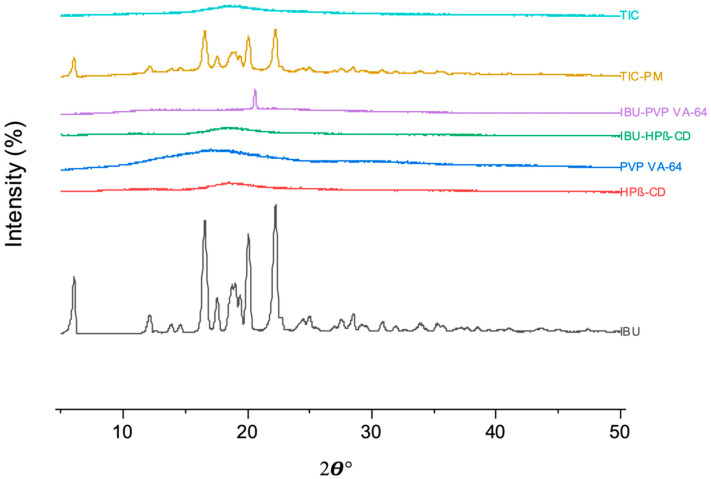
XRD diffractograms of pure IBU, HPβ-CD, PVP VA-64, IBU–HPβ-CD (binary complex), IBU-PVP VA-64 (solid dispersion), TIC-PM (physical mixture), and TIC (ternary inclusion complex).

XRD analysis was used to investigate the drug’s physical state within the extrudates represented in Figure 7. The diffractogram of pure IBU revealed characteristic peaks at 6°, 16.5°, 19.5°, and 22°, indicating the drug’s crystalline nature [48]. The absence of a peak in the HPβ-CD and PVP VA-64 diffractograms indicated their amorphous nature [49,50]. The binary inclusion complex (IBU–HPβ-CD) exhibited no IBU diffraction peaks, indicating that crystalline IBU was converted to an amorphous form due to inclusion complex formation. In contrast, the solid dispersion of IBU-PVP VA-64 showed a characteristic IBU peak at 22°, indicating the presence of crystallinity in the extrudates, which could cause high drug loading. The physical mixture of TIC (TIC-PM) has all the characteristic IBU peaks but with less intensity due to the lower concentration and dilution of IBU in the physical mixture. TIC’s extruded material lacks IBU’s characteristic peaks, indicating that IBU is primarily converted to an amorphous nature through the formation of inclusion complexes and, to a lesser extent, through solid dispersion. In addition, the drug’s enhanced solubility and in vitro dissolution rate further support its conversion to an amorphous form.

### 3.7. NMR

**Figure 8:**
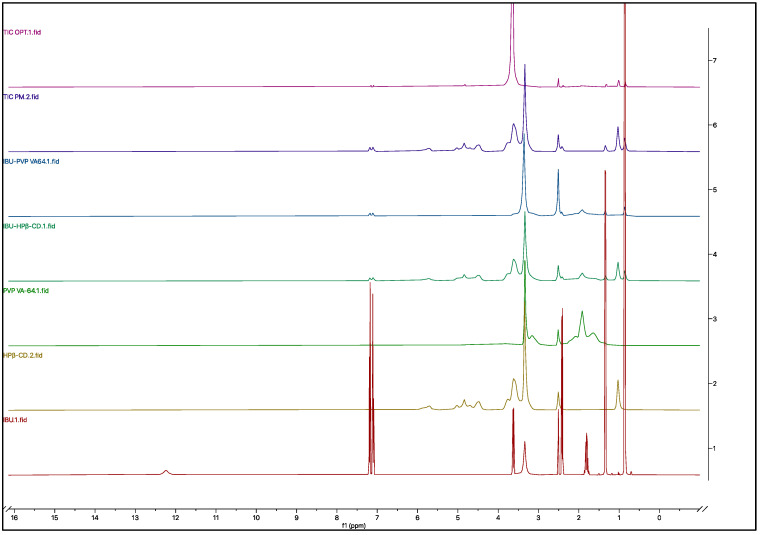
NMR spectrum of pure IBU, HPβ-CD, PVP VA-64, IBU–HPβ-CD (binary complex), IBU-PVP VA-64 (solid dispersion), TIC-PM (physical mixture), and TIC OPT (ternary inclusion complex).

The ^1^H NMR spectroscopies of pure IBU, the excipients, and the formulations are shown in Figure 8. The chemical shifts are denoted in Table S2, and the detailed chemical shift spectrum is represented in Figure S1. The ^1^H NMR spectra of pure IBU displayed peaks at 0.86 (6H, 2CH_3_), 1.34 (3H, CH_3_), 1.81 (1H, CH), 2.41 (2H, CH_2_), 3.63 (1H, CH), 7.11 and 7.19 (4H, aromatic), and 12.24 ppm (1H, COOH) were observed [51]. The chemical shifts were observed in all the formulations, although the carboxylic acid proton peak at 12.24 ppm was not detected in all the formulations (IBU–HPβ-CD, IBU-PVP VA-64, and TIC), which might be due to the hydrogen bond formation with both HPβ-CD and PVP VA-64. In comparison, it can be seen in TIC-PM without any chemical shift. In the case of solid dispersion (IBU-PVP VA-64), no other chemical shifts were observed. Hydrogen bond formation by the carboxylic acid proton was demonstrated in previous studies [52]. The complex formation between IBU and HPβ-CD can be revealed from the significant chemical shift of the 1.81 ppm (1H, CH) and 2.41 ppm (2H, CH_2_) peaks. The exact peak shifts can be observed in TIC, which revealed the hosting of IBU in the hydrophobic cavity of HPβ-CD [46].

### 3.8. Drug Content Uniformity

The drug content of all the samples ranged from 93.6% to 104.2% of labeled claims. According to the USP, a range of 90–110% for the labeled claim is acceptable [53]. Therefore, all the TIC formulations are accepted to be within the specified range of USP.

### 3.9. In Vitro Dissolution Studies

**Figure 9:**
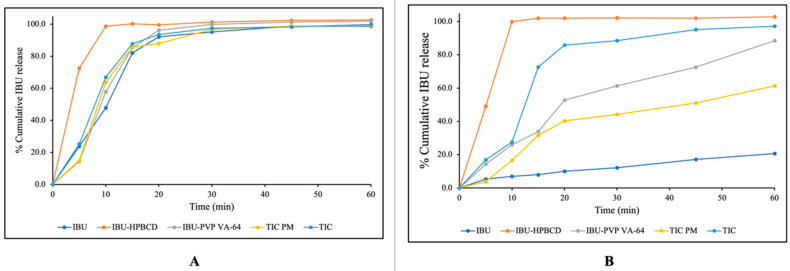
In vitro dissolution profiles of pure IBU and various formulations in pH 7.2 phosphate buffer (**A**) and pH 1.2 0.1 N HCl (**B**), wfor IBU (pure IBU), IBU–HPβ-CD (binary complex), IBU-PVP VA-64 (solid dispersion), TIC-PM (physical mixture), and TIC (ternary inclusion complex).

The dissolution profiles of pure IBU, IBU–HPβ-CD, IBU-PVP VA-64, and TIC are represented in Figure 9. Initially, the dissolution was performed in a pH 7.2 phosphate buffer, and the result indicated that the cumulative drug release was found to be ~85% in 30 min (US FDA 2018 guidance for highly soluble compounds containing immediate release solid oral dosage forms: Q = 80% dissolved in 30 min) [54]. The pH-dependent high solubility of IBU at a basic pH might be the reason behind the faster dissolution profiles of pure IBU, the physical mixtures, and the complexes. The pH 7.2 dissolution media could not discriminate the dissolution rate among the formulations. Therefore, the dissolution media of 0.1 N HCl were used as the discriminatory media, and the trend of drug dissolution in the 0.1 N HCl media was IBU–HPβ-CD (freeze dried) > TIC > TIC-PM > IBU-PVP VA-64 > pure IBU. The cumulative release of pure IBU in 0.1 N HCl was found to be ~20% after 60 min due to the poor solubility of the drug. In the case of the binary inclusion complex, the rapid release was observed to be more than 85% in 15 min, which might be because of the efficient complexation between IBU and HPβ-CD through freeze drying. The rapid release could also be attributed to the large surface area formed by freeze drying due to the high porosity and low density [55]. The release from IBU-PVP VA-64 was higher than that of pure IBU and TIC-PM but was less than that of all other formulations [12]. The dissolution of TIC was found to be more than ~85% in 30 min, which was superior to all other formulations except for the IBU–HPβ-CD binary complex produced by freeze drying. The presence of hydrophilic polymers facilitated and improved the inclusion of IBU in HPβ-CD upon extrusion, which could be one of the reasons for the enhanced dissolution rate by TIC. The improvement in the dissolution of the drug might also be attributed to amorphous IBU in the binary inclusion and TIC, which was verified by ATR, DSC, and XRD studies. The drug release of ~85% in 30 min in both pH 1.2 and pH 7.2 dissolution media signified the high solubility nature of IBU in TIC produced via the hot-melt extrusion process [54]. Table 7 displays the dissolution study findings on pure IBU, TIC-PM, and TIC. The results were compared based on the percentage dissolved after 30 min (PD_30_), the initial dissolution rate (IDR), the dissolution efficiency (DE), and the relative dissolution rate (RDR) after 30 min [56]. It was observed that TIC exhibited seven times greater effectiveness in terms of DE and IDR compared to pure IBU and two times more than TIC-PM.

### 3.10. Stability Studies

The physicochemical changes in formulation upon stability were studied: drug content, dissolution, and the recrystallization of IBU using DSC. The effect of accelerated stability on drug content was very negligible. The dissolution studies carried out in pH 1.2 0.1 N HCl and a pH 7.2 phosphate buffer are given in Figure S2 (Supplementary Files). In pH 1.2 0.1 N HCl dissolution media, the stability sample showed an insignificantly slower release for the initial time points but released more than 85% within the targeted 30 min, while in the case of dissolution in a pH 7.2 phosphate buffer, the dissolution profile of the stability sample was like that of the initial sample. These results demonstrate the similarity of the stability of the sample to that of the initial sample. Thermal studies by DSC, as represented in Figure S3 (Supplementary File), revealed the absence of the recrystallization of IBU after being subjected to accelerated stability studies for 3 months.

## 4. Conclusions

This study evaluated five different types of CDs for their suitability to form inclusion complexes with IBU by in silico and in vitro techniques. The results indicated that HPβ-CD formed a complex with IBU in a 1:1 equimolar stoichiometric ratio. To achieve continuous complex formation, HME was employed, and a polymer was added as an auxiliary substance. Quality by design (QbD) principles were utilized to investigate the impact of independent parameters such as the barrel temperature, screw speed, and polymer concentration on dependent variables like IBU solubility and dissolution. The successful formation of the complex was confirmed after extrusion through HME, resulting in a significantly improved saturation solubility of IBU compared to that of the physical mixtures. Dissolution testing in pH 1.2 0.1 N HCl and a pH 7.2 phosphate buffer revealed the enhanced dissolution of the extruded formulations in pH 1.2 0.1 N HCl compared to the physical mixtures and solid dispersions (IBU-PVP VA-64). The release rate of the extruded formulations was slower than that of the binary complex (IBU–HPβ-CD) formed by freeze drying. Still, it achieved ≥85% release within 30 min, indicating the high solubility of IBU through complex formation via the extrusion process. Analytical techniques, including DSC, XRD, ATR, and NMR, confirmed the amorphous nature of IBU in TIC and the formation of the inclusion complex between IBU and HPβ-CD. Overall, this study emphasized the importance of the polymer matrix and rheology, influenced by the polymer concentration and barrel temperature, in facilitating TIC formation through the extrusion process. When the temperature rose, the viscosity of the polymer decreased. This allowed the drug to mix or easily suspend within the polymer matrix and then enter the hydrophobic cavity of cyclodextrins. Polymer concentration played a critical role in providing viscosity for extrusion while maintaining an appropriate formulation bulkiness. The screw speed was adjusted to ensure smooth processability by minimizing torque. In culmination, this study not only advanced our comprehension of TIC formation via extrusion but also served as a paradigm of comprehensive pharmaceutical manufacturing. By amalgamating rheological insights, formulation variables, and a quality by design perspective, the research elucidated a path for manufacturing techniques. Additionally, such systems could potentially alleviate the solubility challenges of poorly soluble drugs, potentially enhancing their therapeutic efficacy and overall bioavailability.

## Figures and Tables

**Table 1:** Independents variables with their coded levels for Box–Behnken design.

Independent Variables	Coded Levels		
	–1	0	+1
PVP VA-64 (X_1_, % *w*/*w*)	10	20	30
Extrusion temperature (X_2_, °C)	115	130	145
Screw speed (X_3_, rpm)	15	30	45

**Table 2:** Box–Behnken design parameters for various experimental runs and observed values of solubility and release of hot-melt extruded ibuprofen TIC.

Run *	Assigned Independent Variables			Actual Independent Variables			Response	
	X_1_	X_2_	X_3_	PVP VA-64 (% *w*/*w*)	Extrusion Temp (°C)	Screw Speed (rpm)	Solubility (µg/mL)	Release after 30 min (%)
1	0	0	0	20	130	30	5131	78.1
2	+1	+1	0	30	145	30	2586	60
3	0	0	0	20	130	30	5132	78
4	0	−1	+1	20	115	45	Extrusion Failure	
5	0	0	0	20	130	30	5130	77.9
6	−1	0	−1	10	130	15	3238	87
7	0	−1	−1	20	115	15	Extrusion Failure	
8	0	0	0	20	130	30	5129	78.3
9	0	0	0	20	130	30	5133	77.8
10	0	+1	−1	20	145	15	4755	70
11	0	+1	+1	20	145	45	4723	95
12	+1	−1	0	30	115	30	Extrusion Failure	
13	+1	0	+1	30	130	45	5703	84
14	−1	−1	0	10	115	30	4071	85
15	+1	0	−1	30	130	15	3342	94
16	−1	0	+1	10	130	45	4297	78
17	−1	+1	+1	10	145	30	3907	85

* All runs contain IBU:HPβ-CD (1:1) stoichiometric ratio.

**Table 3:** Software data obtained from analysis of variance testing for the selected quadratic model of Box–Behnken design for the optimization process of solubility and release of hot-melt extruded TIC.

Source	Solubility (X_1_, µg/mL)				Release after 30 min (X_2,_ %)			
	Sum of Squares	DF	F-Value	*p*-Value	Sum of Squares	DF	F-Value	*p*-Value
Model	1.069 × 10^7^	9	4.751 × 10^5^	<0.0001	1054.92	9	3167.91	<0.0001
A	5.700 × 10^5^	1	2.280 × 10^5^	<0.0001	42.25	1	1141.89	<0.0001
B	1.673 × 10^6^	1	6.690 × 10^5^	<0.0001	330.75	1	8939.19	<0.0001
C	2.924 × 10^6^	1	1.170 × 10^6^	<0.0001	90.25	1	2439.19	<0.0001
AB	1.437 × 10^6^	1	5.746 × 10^5^	<0.0001	330.75	1	8939.19	<0.0001
AC	4.238 × 10^5^	1	1.695 × 10^5^	<0.0001	0.2500	1	6.76	0.0601
BC	1.012 × 10^6^	1	4.046 × 10^5^	<0.0001	396.75	1	10,722.9	<0.0001
A^2^	4.237 × 10^6^	1	1.695 × 10^6^	<0.0001	3.55	1	96.05	0.0006
B^2^	2.613 × 10^5^	1	1.045 × 10^5^	<0.0001	149.75	1	4047.42	<0.0001
C^2^	1.769 × 10^5^	1	70,770.28	<0.0001	216.80	1	5859.33	<0.0001
Pure error	10.00	4	2.50		0.1480	4	0.0370	
Cor total	1.069 × 10^7^	13			1055.06	13		
Sum of squares	Type III Partial				Type III Partial			
Model F-value	475,081.48				3167.91			
R^2^	1.0				0.9999			
Adjusted R2	1.0				0.9995			
Adequate precision	2332.5492				215.294			
Standard deviation	1.58				0.1924			
Mean	4448.36				80.58			
C.V. (%)	0.0355				0.2387			

***p*-values** lower than 0.05 indicate significant model terms, and values greater than 0.1000 indicate that the model terms are insignificant; **DF**; degree of freedom; **C.V.**; coefficient of variation.

**Table 4:** Variables’ criteria and responses for the optimization process.

Variable	Goal	Lower Limit	Upper Limit
PVP VA-64 (X_1_, % *w*/*w*)	minimize	20	30
Extrusion temperature (X_2_, °C)	maximize	130	145
Screw speed (X_3_, rpm)	in range	15	45
Solubility (mg/mL)	maximize	4500	6000
Release after 30 min	in range	85	100

**Table 5:** Software-proposed solutions during the optimization step.

Trial No.	Kollidon VA 64 (% *w*/*w*)	Extrusion Temperature (°C)	Screw Speed (rpm)	Solubility (µg/mL)	Release after 30 min (%)	Desirability
**1**	**20.000**	**138.129**	**45.000**	**5299.706**	**86.286**	**0.661**
2	20.002	137.500	45.000	5362.453	85.726	0.661
3	20.009	137.822	44.910	5324.795	85.871	0.660
4	20.000	138.077	44.862	5296.628	86.033	0.656
5	20.000	138.092	44.708	5286.053	85.821	0.647
6	20.822	138.373	45.000	5277.603	86.029	0.635
7	20.000	138.728	43.751	5172.099	85.000	0.632
8	20.987	137.242	44.970	5394.529	85.000	0.622
9	20.000	139.206	43.453	5113.537	85.000	0.606
10	20.771	144.523	45.000	4725.967	93.290	0.463
11	23.841	139.802	45.000	4967.657	85.000	0.271
12	24.210	142.726	45.000	4597.360	87.025	0.164
13	20.000	131.250	18.094	4579.473	85.000	0.164
14	20.160	131.232	18.153	4581.665	85.000	0.164
15	20.000	131.146	18.259	4586.201	85.000	0.163
16	20.216	131.200	18.214	4584.009	85.000	0.163
17	20.365	131.269	18.130	4579.487	85.000	0.161
18	20.454	131.175	18.299	4585.922	85.000	0.152
19	20.621	131.296	18.129	4576.392	85.000	0.150
20	20.234	130.759	18.933	4616.398	85.000	0.128
21	20.000	132.762	15.751	4509.555	85.000	0.111
22	20.000	130.023	20.078	4675.437	85.000	0.093

**Table 6:** Results of validation trials of the software-suggested solution (mean, n = 3).

Response	Software-Predicted Values	Standard Deviation	Standard Error	Observed Values	95% CI Low for Mean	95% CI High for Mean
Solubility (µg/mL)	5299.71	1.58114	1.1503	5292.59	5287.59	5311.82
Release after 30 min	86.286	0.192354	0.13994	86.5	84.8117	87.7593

**Table 7:** Percentage of drug dissolved after 30 min (PD), initial dissolution rate (IDR), dissolution efficiency (DE), and relative dissolution rate (RDR) of pure IBU, TIC-PM, and TIC.

Formulation	PD_30_	IDR (%/min)	DE_30_ (%)	RDR_30_
IBU	12.10	0.40	7.88	--
TIC-PM	44.20	1.47	26.07	3.65
TIC	88.50	2.95	55.68	7.31

## Data Availability

The data presented in this study are available in this article.

## Supplementary Materials

The following supporting information can be downloaded at: https://www.mdpi.com/article/10.3390/pharmaceutics15092203/s1, Figure S1: Detailed Chemical shifts in NMR spectrum of pure IBU and formulations; Figure S2: In vitro dissolution profiles of Pure IBU and various formulations in pH 7.2 phosphate buffer (A) and pH 1.2 0.1N HCl (B); Figure S3: DSC thermograms of Pure IBU and formulations after 3 months of stability at 40 ± 2 °C; Table S1: The Ks and CE of all the cyclodextrins; Table S2: ^1^H Nuclear Magnetic Resonance (^1^H NMR) Chemical Shift Data.
