# Supplementary material for: Continuous Manufacturing of Solvent-Free Cyclodextrin Inclusion Complexes for Enhanced Drug Solubility via Hot-Melt Extrusion: A Quality by Design Approach
Source: Pharmaceutics. 2023 Aug 25;15(9):2203. doi: 10.3390/pharmaceutics15092203 (PMC10536280; doi:10.3390/pharmaceutics15092203)
Supplement: Supplementary file 1 [file pharmaceutics-15-02203-s001.zip › pharmaceutics-2541534-supplementary.pdf]

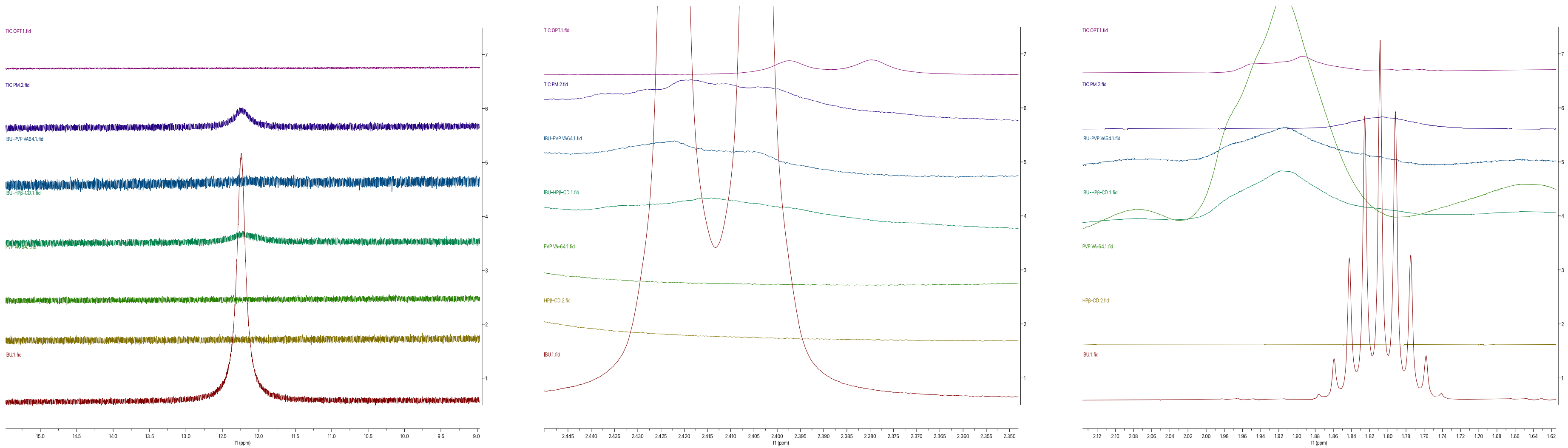

Figure S1: Detailed Chemical shifts in NMR spectrum of pure IBU and formulations

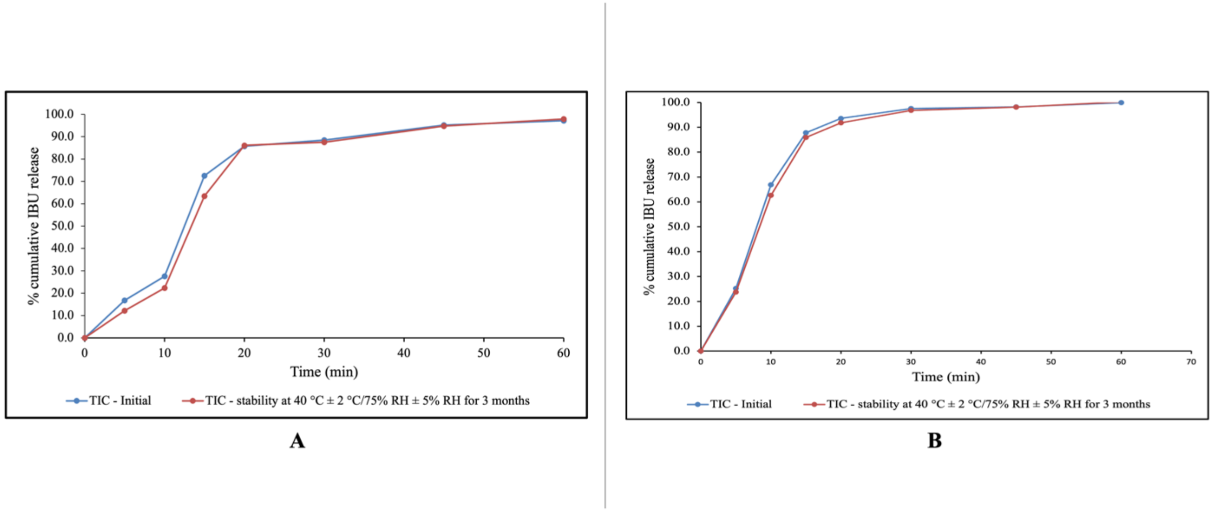

Figure S2: In vitro dissolution profiles of Pure IBU and various formulations in pH 7.2 phosphate buffer (A) and pH 1.2 0.1N HCl (B)

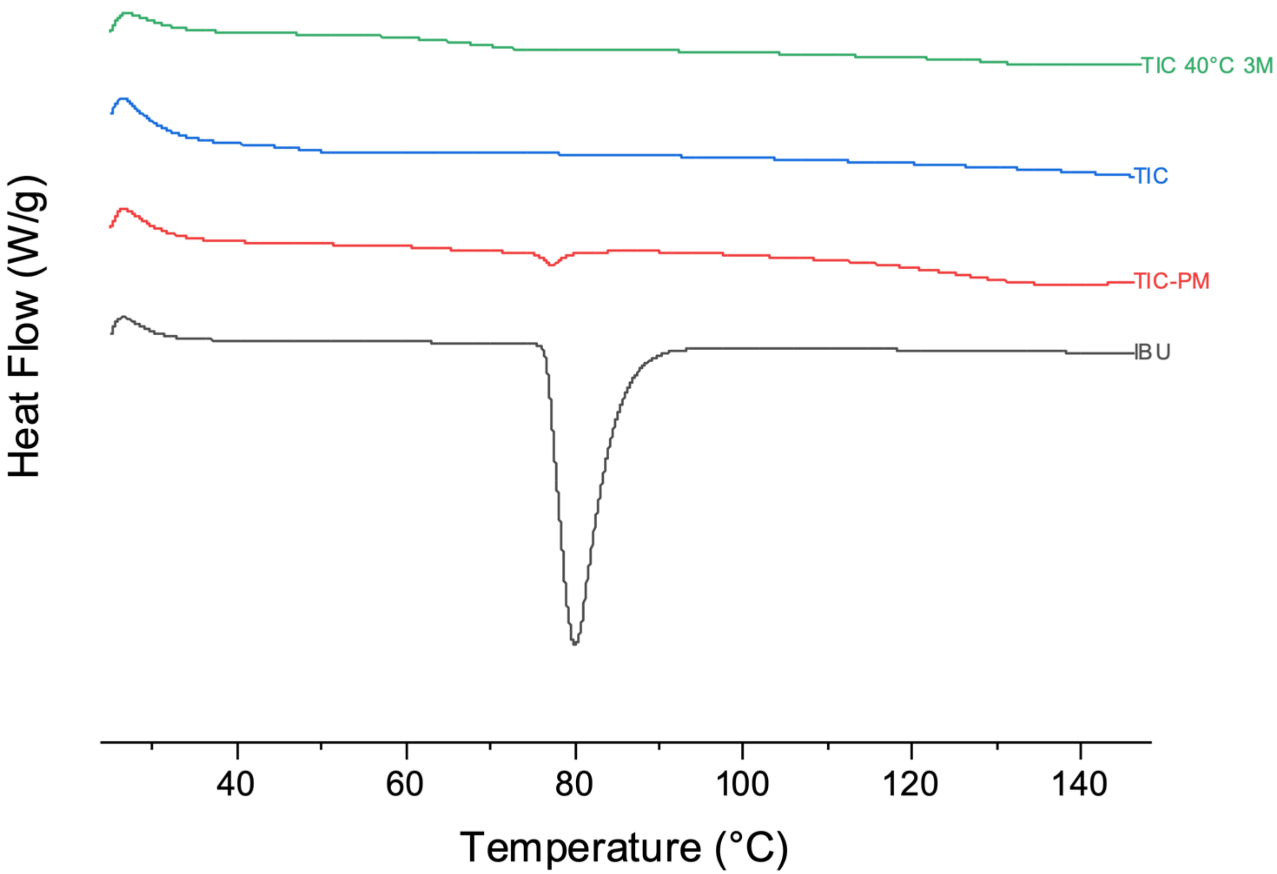

Figure S3: DSC thermograms of Pure IBU and formulations after 3months of stability at 40°C ± 2°C

Table S1: The Ks and CE of all the cyclodextrins.

**Cyclodextrin**	**Ks (M)**	**CE**
α-CD	75.85	0.019
β-CD	365.32	0.091
γ-CD	100.11	0.025
HPβ-CD	3279.62	0.820
HPγ-CD	177.37	0.044

Table S2: ^{1}H Nuclear Magnetic Resonance (1H NMR) Chemical Shift Data

Proton	IBU	HPBCD	PVPVA-64	IBU-HPBCD	ΔδA	IBU-PVPVA64	ΔδB	TIC-PM	ΔδC	TIC OPT	ΔδD
1	**12.24**	--	--		**ND**		**ND**	12.23	-0.01		**ND**
2	**7.19**	--	--	7.18	-0.01	7.185	0.00	7.19	0.00	7.16	-0.03
3	**7.11**	--	--	7.1	-0.01	7.1	-0.01	7.11	0.00	7.08	-0.03
4	--	5.72	--	5.72	NA		--	5.72	NA	5.82	NA
5	--	4.84	--	4.84	NA		--	4.84	NA	4.82	NA
6	--	4.5	4.53	4.5	NA		NA	4.5	NA		NA
7	--	--	3.98		--		NA		NA		NA
8	--	--	3.76		--		NA		NA		NA
9	**3.63**	--	--	3.61	-0.01	3.62	0.00	3.64	0.02	3.65	0.03
10	--	3.36	3.33		NA		NA		NA		NA
11	**2.41**	--	--	2.51	**0.10**	2.42	0.01	2.42	0.01	2.51	**0.10**

12	--	--	2.09		--		NA		NA		NA
13	--	--	1.91		--	1.91	NA		NA	1.92	NA
14	**1.81**	--	--	1.91	**0.10**	1.8	-0.01	1.81	0.00	1.89	**0.08**
15	--	--	1.64		--		NA		NA		NA
16	**1.34**	--	--	1.33	-0.01	1.34	0.00	1.33	-0.01	1.32	-0.02
17	--	1.03	--	1.03	NA		--	1.03	NA	1.01	NA
18	**0.86**	--	--	0.85	-0.01	0.86571429	0.01	0.86	0.00	0.83	-0.03
